# Latitudinal diversity gradients in Mesozoic non-marine turtles

**DOI:** 10.1098/rsos.160581

**Published:** 2016-11-23

**Authors:** David B. Nicholson, Patricia A. Holroyd, Paul Valdes, Paul M. Barrett

**Affiliations:** 1 Department of Earth Sciences, The Natural History Museum, Cromwell Road, London SW7 5BD, UK; 2 Museum of Paleontology, University of California, 1101 Valley Life Sciences Building, Berkeley, CA 94720, USA; 3 School of Geographical Sciences, University of Bristol, University Road, Bristol BS8 1SS, UK

**Keywords:** latitudinal biodiversity gradient, Testudinata, subsampling, genus richness, geographical range

## Abstract

The latitudinal biodiversity gradient (LBG)—the pattern of increasing taxonomic richness with decreasing latitude—is prevalent in the structure of the modern biota. However, some freshwater taxa show peak richness at mid-latitudes; for example, extant Testudines (turtles, terrapins and tortoises) exhibit their greatest diversity at 25° N, a pattern sometimes attributed to recent bursts of climatically mediated species diversification. Here, we test whether this pattern also characterizes the Mesozoic distribution of turtles, to determine whether it was established during either their initial diversification or as a more modern phenomenon. Using global occurrence data for non-marine testudinate genera, we find that subsampled richness peaks at palaeolatitudes of 15–30° N in the Jurassic, 30–45° N through the Cretaceous to the Campanian, and from 30° to 60° N in the Maastrichtian. The absence of a significant diversity peak in southern latitudes is consistent with results from climatic models and turtle niche modelling that demonstrate a dearth of suitable turtle habitat in Gondwana during the Jurassic and Late Cretaceous. Our analyses confirm that the modern testudinate LBG has a deep-time origin and further demonstrate that LBGs are not always expressed as a smooth, equator-to-pole distribution.

## 1. Introduction

The modern latitudinal biodiversity gradient (LBG), with a tropical peak in species richness that declines polewards, is the predominant biogeographical pattern on the Earth [1,2]. It shows remarkable consistency across taxonomic groups and habitats [1,2] and exceptions are rare (e.g. [3]). Although this fundamental and global macroecological pattern has been assumed to be conserved through deep time, recent analyses of Mesozoic and Cenozoic organismal distributions have indicated that this may not be the case [4]. A modern-type LBG can only be identified unequivocally during several Palaeozoic intervals and over the past 30 Myr [4]: indeed, that of Cenozoic North American land mammals has been established for only 4 Myr [5]. In all cases, it is generally associated with global icehouse conditions [4,5].

Extant Testudines (turtles, terrapins and tortoises, referred to as turtles collectively herein) are an exception to the modern LBG. This clade shows a complex, asymmetric pattern with a pronounced diversity peak at 25° N and the absence of a major peak in the Southern Hemisphere [6,7]. It has been posited that this Northern Hemisphere peak in richness is a consequence of either an underlying pattern of decreasing range size at lower latitudes in combination with geographical centre of origin at these latitudes [7], or a set of complex correlations among temperature, land area, and phylogenetic and biogeographic history [6]. However, modern data do not allow us to choose between these alternatives. Moreover, prior studies of turtle latitudinal distributions have not attempted to determine whether this pattern has a deep-time origin or whether it is a consequence of more recent climatic and geographical factors.

Mapping fossil occurrences, placing them within their environmental contexts and assessing species richness within latitudinal bins through time (usually using various forms of subsampling methods) have all been used to test for the existence of a modern-type LBG in deep time [8]. Here, we analyse an occurrence dataset of Mesozoic non-marine testudinate genera to test two hypotheses: (i) Did Mesozoic turtle genus richness vary with palaeolatitude through time?; and, if so, (ii) Was a modern-type LBG established in this earlier greenhouse world? We also compare testudinate latitudinal distributions with those previously published for other Mesozoic vertebrates (dinosaurs [9] and pseudosuchians [10]) and discuss potential factors that may have influenced these patterns. Understanding the processes governing biogeographic distributions over extended spatial and temporal scales, which are accessible only via the fossil record, can help us to quantify the likely risks of and responses of extant species to contemporary climate change, in terms of identifying possible future extinctions, extirpations, range changes and changes to adaptations in niche exploitation [11–15].

## 2. Methods

### 2.1. Data

Following a period of additional data entry and revision by the authors, data on fossil turtle occurrences were drawn from the Paleobiology Database (PBDB; www.paleobiodb.org) via the Fossilworks portal (http://fossilworks.org) on 2 August 2016 using the search terms Testudinata and Mesozoic, comprising 1862 occurrences of 271 genera in 1103 PBDB collections (electronic supplementary material, Dataset S1, sheet 1) [16]. Occurrences of marine taxa, ootaxa (fossil eggs) and other ichnotaxa (as listed in [17]) were removed, leaving 1506 occurrences of 192 genera in 894 PBDB collections (electronic supplementary material, Dataset S1, sheet 2). Two temporal binning schemes were used. For both raw and subsampled richness estimates, data were partitioned into 15° palaeolatitudinal bands for the Maastrichtian (Maas), Campanian (Camp), Berriasian–Santonian (rCret), Jurassic (Jur) and Triassic (Tr) bins. Palaeolatitudinal estimates were generated by the Fossilworks website from the present-day coordinates of each collection, based on tectonic plate rotations from the PALEOMAP Project (www.scotese.com). Unique genera were counted for each bin. We selected genera in order to use the most stable taxonomy while maximizing the available occurrences. Palaeolatitudinal bands of 15° were the smallest feasible geographical divisions for statistically meaningful sample sizes per bin. A second binning scheme for the generalized least-squares (GLS) regressions (see below) split the data into Jurassic, Early Cretaceous and Late Cretaceous. Unique turtle-bearing formation (TurtBF) names were counted for each bin. Where occurrences did not have a named formation, the group name was inserted instead if this was certain not to artificially inflate the formation count (i.e. other named formations from that group were not in the dataset), or by the collection/locality name if no other deposits from that age or geographical area existed in the dataset, with justification for these decisions noted in electronic supplementary material, Dataset S1. A separate download of Mesozoic Tetrapoda, similarly limited to include only non-marine taxon occurrences and exclude ichnotaxa (as used in [18]), provided counts of tetrapod-bearing collections (TetBC) for the GLS regressions. It is worth noting that, in the Mesozoic, with the exception of the now-extinct Nanhsiungchelyidae and some other stem-turtles, non-marine turtles were largely associated with freshwater habitats, with the origin of modern land tortoises (Testudinidae) in the Palaeogene [19].

Non-marine area (NMA), i.e. the area comprising both land and terrestrial freshwater aquatic ecosystems, was calculated in 5° latitudinal bands from a set of palaeogeographic reconstructions from each relevant geological stage. These reconstructions used the same methodology as previous work [20]. These 5° latitudinal bands were combined into 15° bands and the geometric mean of the land area calculated for each of the time bins analysed in the GLS (electronic supplementary material, Dataset S1, sheet 4 and Dataset S2).

Data on the latitudinal distribution of extant turtle species were taken from Angielczyk *et al.* [6]. These data were re-binned from species to genus level and from 5° to 15° latitudinal bins, in order to match the binning strategy used for the fossil turtles. From these data a new statistic, number of genera divided by log_10_ non-marine area per latitudinal band, was calculated as an alternative way of measuring richness per unit area and that can be used to compare with fossil richness and distribution data.

### 2.2. Analyses

Raw genus richness and subsampled genus richness was calculated for each time bin (Tr, Jur, rCret, Camp, Maas) and each 15° latitudinal band. Subsampled richness was estimated with Shareholder Quorum Subsampling (SQS) [21], using Perl script version 4.3, available in the supplementary material of Benson *et al.* [17], in order to account for unevenness in sampling.

Multiple generalized least-squares regression models were calculated using the gls() function of the nlme package [22] in R [23]. The response variable of raw counts of genus richness through latitude for each time bin (Jurassic, Early Cretaceous and Late Cretaceous) was analysed in the following combinations: (i) turtle-bearing formation counts (TurtBF), used here as a proxy for the diversity of available habitats, as the lithologies and sedimentary features representing different depositional settings in different areas are typically recognized by different formation names; (ii) counts of non-marine tetrapod-bearing collections (TetBC), a proxy for sampling opportunity, showing the potential for fossil bone of any type to be recovered from a particular locality [24]; (iii) non-marine area (NMA), a proxy for the extent of freshwater aquatic and terrestrial areas potentially available as turtle habitat and reflecting the changing distribution and extent of land masses through time; (iv) TurtBF + NMA; (v) TetBC + NMA and (vi) an intercept-only null model. To determine the best predictors, a preferred model from each set was identified by the second-order Akaike information criterion (AICc) weight, which contains a correction for small sample sizes [25,26] using the aictab() function of the AICcmodavg package [27] in R. Model *p*-values were computed by comparing the model with the null model for the response variable using an ANOVA. The *R*^2^ reported is the generalized *R*^2^ of Nagelkerke [28], calculated manually from the GLS output of the models and null models, using the formula expressed in the supplementary information of Mannion *et al.* [9].

## 3. Results

**Figure 1.**
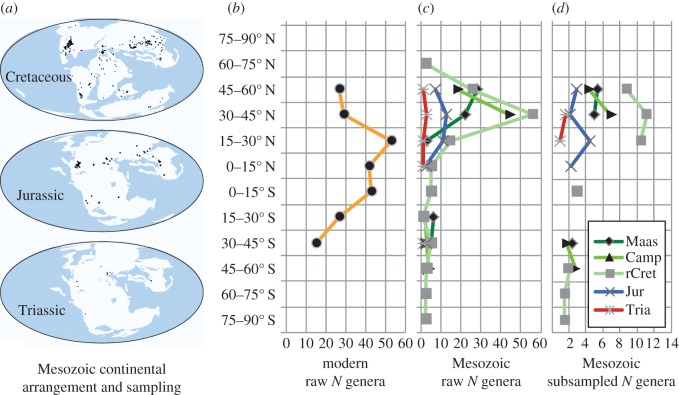
Latitudinal generic richness of non-marine turtles in the Recent and Mesozoic. (*a*) Palaeocontinental reconstructions from http://fossilworks.org [29]. Black dots indicate the positions of non-marine turtle-bearing collections in the database. (*b*) Raw counts of Recent non-marine turtle genera by latitude, based on data from [6]. (*c*) Raw counts of Mesozoic non-marine turtle genera by latitude. (*d*) Subsampled estimates of relative non-marine turtle generic richness using Shareholder Quorum Subsampling (SQS). Maas, Maastrichtian; Camp, Campanian; rCret, Berriasian–Santonian; Jur, Jurassic; Tria, Triassic. N.B. As these time bins vary greatly in length, it is inappropriate to compare values between them. Values should only be compared across latitude within each time bin.

**Figure 2.**
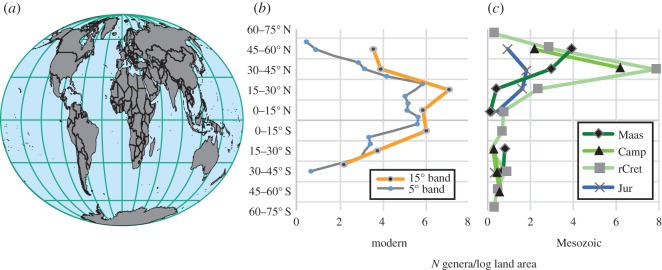
Raw counts of non-marine turtle genera by latitude divided by log_10_ land area. (*a*) Recent global continental configuration modified from http://fossilworks.org [29]. (*b*) Recent data from [6], taken at genus level and re-binned at 5° (blue dots) and 15° (black dots) latitudinal bands, and divided by Recent log_10_ land area. (*c*) Mesozoic non-marine turtle genera by 15° latitudinal bands, and divided by log_10_ palaeo-land area. Abbreviations as in figure 1.

The palaeolatitudinal distribution of Mesozoic turtles extends both further north and south than today, from approximately 72° N to 76° S (figures 1 and 2). As previously reported [18], the absence of turtles from the highest northern palaeolatitudes (from which other tetrapods have been sampled) indicates real extrinsic limits on their distribution rather than a sampling bias. Raw genus richness peaks at 30–45° N in all time bins except the Maastrichtian, which has peak richness at 45–60° N (figure 1*c*). A similar pattern is recovered from subsampled richness, with the exception that the Jurassic peak occurs further south at 15–30° N (figure 1*d*). The Southern Hemisphere contributes fewer occurrences to the dataset than the Northern Hemisphere in all time bins (figure 1*a*) with very low numbers of genera and an essentially flat LBG.

Generalized least-squares (GLS) regressions on observed genus counts per latitudinal band for the Early and Late Cretaceous indicate that different models explain the observed distributional patterns at different times (table 1). For the Jurassic, the null (intercept-only) model is preferred with a sample size corrected Akaike Information Criterion (AICc) weight of 0.98 (*R*^2^ = 0.947, *p* < 0.0001), despite generalized *R*^2^ values of 0.915 and 0.683 for the turtle-bearing formations (TurtBF) and tetrapod-bearing collections (TetBC) models, respectively. The Jurassic non-marine area (NMA) model performed very poorly (generalized *R*^2^ = 0.002) and the AICc of the TurtBF + NMA and TetBC + NMA models was incalculable, due to the number of parameters being one less than the number of observation points. For the Early Cretaceous the TurtBF model is strongly preferred, with an AICc weight of 0.94 and generalized *R*^2^ of 0.931, and second preference given to the TetBC + NMA model (AICc weight = 0.04) which, despite having a higher *R*^2^ value (0.937), was heavily penalized for over-fitting. For the Late Cretaceous the marginally preferred model is TetBC (AICc weight = 0.78, *R*^2^ = 0.948) with TurtBF in second place (AICc weight = 0.18, *R*^2^ = 0.924, *p* < 0.0001). Between time bins, TetBC had lowest significance as an explanatory variable in the Jurassic (*R*^2^ = 0.683, *p* = 0.0165), high significance in the Early Cretaceous (*R*^2^ = 0.839, *p* = 0.0001) and greatest significance in the Late Cretaceous (see above). The NMA-only models performed poorly in all time bins.

**Table 1.** Comparison of generalized least-squares (GLS) regression models for observed non-marine turtle genus richness in 15° latitudinal bands for the Late Cretaceous, Early Cretaceous and Jurassic. TurtBF, non-marine turtle-bearing formations; TetBC, non-marine tetrapod-bearing collections; NMA, non-marine area; *R*^2^, generalized *R*^2^; AICc, second-order Akaike information criterion; *w_i_*, AICc weight; LL, log-likelihood.

model sets	*R*^2^	*p-*value	AICc	ΔAICc	*w_i_*	LL
Late Cretaceous						
genus diversity ∼ TetBC	0.948	<0.0001	0.15	0.00	0.78	5.92
genus diversity ∼ TurtBF	0.924	<0.0001	3.11	2.95	0.18	4.45
genus diversity ∼ TetBC + NMA	0.965	<0.0001	6.29	6.14	0.04	7.52
genus diversity ∼ TurtBF + NMA	0.924	<0.0001	12.44	12.29	0.00	4.45
null	—	—	18.14	17.99	0.00	−5.87
genus diversity ∼ NMA	0.086	0.3978	23.02	22.87	0.00	−5.51
Early Cretaceous						
genus diversity ∼ TurtBF	0.931	<0.0001	−0.66	0.00	0.94	5.73
genus diversity ∼ TurtBF + NMA	0.937	<0.0001	5.79	6.45	0.04	6.10
genus diversity ∼ TetBC	0.839	0.0001	7.01	7.67	0.02	1.89
genus diversity ∼ TetBC + NMA	0.868	0.0001	12.37	13.03	0.00	2.81
null	—	—	18.62	19.28	0.00	−6.31
genus diversity ∼ NMA	0.407	0.0301	18.72	19.38	0.00	−3.96
Jurassic						
null	—	—	15.22	0.00	0.98	−2.61
genus diversity ∼ TurtBF	0.915	0.0005	22.92	7.70	0.02	3.54
genus diversity ∼ TetBC	0.683	0.0165	29.48	14.26	0.00	0.26
genus diversity ∼ NMA	0.002	0.919	35.21	19.99	0.00	−2.61
genus diversity ∼ TurtBF + NMA	0.915	0.0021	∞	∞	0.00	3.54
genus diversity ∼ TetBC + NMA	0.69	0.0534	∞	∞	0.00	0.32

## 4. Discussion

In the Mesozoic greenhouse world, non-marine turtles ranged further north and south than in the modern day (see also [18]). Triassic turtles achieved a worldwide distribution, but are too rare to reveal any latitudinal patterns. By contrast, Jurassic turtles show contrasting peaks between raw (30–45° N) and subsampled (15–30° N) genus richness, but exhibit the earliest evidence of a mid-latitude, Northern Hemisphere diversity peak. In the Cretaceous, the peak in both raw and subsampled generic richness was further north at 30–45° N (rather than 15–30° N as seen today [6]), and the raw data suggest that it may have shifted even further north to 45–60° N during the Maastrichtian although this peak is less pronounced in the subsampled data (figure 1*c*). These results are consistent with other studies that have demonstrated that LBGs are not constant through time, but are dynamic patterns that are strongly affected by changes in global climatic regimes [4,15]. They cannot be regarded as fixed global patterns for all taxa, contrary to widely held assumptions, nor can we expect future LBGs to necessarily retain a tropical to subtropical peak [4], within the climatic constraints imposed by the biological limitations of ectothermy and obligate ovipary [30]. Similar to the pattern in extant turtles [6], we found a global diversity peak at mid-latitudes in the Northern Hemisphere. The subtle shift of the peak to 45–60° N in the Maastrichtian subsampled richness coincides with a well-documented northward expansion of taxa in North America at this time [31]. This range expansion might have been driven by short-term continental scale temperature increases from the Early to Late Maastrichtian that enabled some turtles to increase their ranges [31,32]. The persistence of peak richness at mid-latitudes in the Northern Hemisphere throughout the Mesozoic, despite turtles having a wider latitudinal range than at present [18], and the evidence for a propensity for range expansion in response to climate change in both the Cretaceous [31] and more recent times [15], suggests that persistent abiotic mechanisms control these latitudinal patterns.

Differences in sampling intensity among regions may influence the patterns recovered by a global analysis; for example, it is plausible that the mid-latitude northern peak might be driven primarily by the fossil record from the more intensively sampled North American land mass in which the most occurrences are recorded. However, the eastern half of the Northern Hemisphere contributes more genera (Triassic, *n* = 4; Jurassic, *n* = 20; Cretaceous, *n* = 90) to the dataset than the western half (Triassic, *n* = 2; Jurassic, *n* = 7; Cretaceous, *n* = 54), so the peaks we find in taxonomic richness are not driven simply by the land mass with most occurrences. Similarly, it may be tempting to attribute the absence of a Southern Hemispheric peak in genus richness to the smaller number of occurrences available, even though the modern turtle LBG also lacks a southern peak [6]. However, climatic niche modelling indicates that, at least during the Maastrichtian, habitats with the combination of temperature and precipitation favoured by turtles were rare in Gondwana [33]. Moreover, during the Late Jurassic, large swathes of the Gondwanan mid-latitudes were covered by desert [34], which is also incompatible with high turtle diversity. This lack of suitable ecospace suggests that the absence of a Southern Hemispheric richness peak might be a genuine signal, controlled by biological and environmental factors, rather than a sampling issue.

Determining the factors that best explain the patterns observed in the turtle data revealed that, in strong contrast with previous work on Mesozoic dinosaurs [9], non-marine area did not contribute to the best predictive models (table 1). The comparative lack of importance of non-marine area probably reflects the fact that it is a poor proxy measure for available turtle habitats: indeed, dividing the raw counts of genera by log_10_ non-marine area has negligible effect on the genus richness profile in either the Recent or Mesozoic (figure 2). Many Mesozoic non-marine turtles are freshwater aquatic and some are inferred to be obligately aquatic [35]. Today, freshwater ecosystems comprise only a small proportion of total non-marine land area, with estimates ranging from approximately 0.8% to approximately 3% of the Earth's surface [36]. Areas of greatest taxonomic richness for extant turtles are associated with extensive river/wetland habitats [37], and several studies of other aquatic vertebrates have shown only partial concordance between areas of high terrestrial and high aquatic richness [38].

Extant crocodylians and their extinct relatives have an extensive fossil record and are potentially better ecological analogues for turtles than dinosaurs; like turtles, many members of this clade are freshwater aquatic and all are ectotherms. Mannion *et al*. [10] recovered a tropical peak in subsampled pseudosuchian genus richness in the Triassic and evidence of high diversity in individual low-palaeolatitude formations during the Cretaceous, a pattern that is replicated today. The strong contrast between the pseudosuchian LBG and that of both Mesozoic and extant turtles may be due, in part, to greater morphological and ecological variation among Mesozoic than Recent pseudosuchians, as some extinct members of the clade (e.g. notosuchians, protosuchians) exploited fully terrestrial niches [39,40], or to different climatic preferences between pseudosuchians and turtles [41].

The association between high modern turtle taxic richness and large river systems may explain, in part, our finding that the number of turtle-bearing formations is one of the best predictors of past taxonomic richness. Terrestrial geological formations typically comprise rocks within a single sedimentary basin that correspond to ancient watersheds. Therefore, as we sample more formations, we also sample more ancient watersheds and would expect taxonomic richness to increase (as the number of watersheds is a key predictor for modern turtle richness [6]). However, it is difficult to say whether this effect is eclipsed by the well-known covariance of fossil-bearing formations and taxonomic richness in the fossil record [42], which may be indicated by the strong predictive power of the sampling proxy (tetrapod-bearing collections) in the Early and Late Cretaceous models.

In modern biomes, LBGs for taxa associated with freshwater habitats are generally less steep than those found in terrestrial or marine habitats [1,43]. These differences may be attributable to the more even distribution of freshwater biome area between temperate and tropical zones [1,44] or greater concentration of taxonomic richness in key freshwater ecoregions [38]. Furthermore, several aquatic vertebrate and invertebrate groups depart from the expected pattern of high low-latitude richness, showing either flat diversity (Caudata and Trichoptera [45]), greater richness in temperate regions (Ephemeroptera, Plecoptera, amphipod crustaceans, shredder detritivores [45,46]) or other complex patterns (aquatic plants [43]).

Although current data cannot be used to test all of the hypotheses proposed for the formation of the modern turtle LBG [6,7], these Mesozoic data do allow us to eliminate some suggested mechanisms. Our finding that richness is not a function of land area allows us to reject hypotheses that suggest that the modern gradient is a simple function of greater geographical area in the tropics [43]. Rather the more equable climates of the Mesozoic allowed expansion of the latitudinal range of turtles [18] and probably led to novel combinations of the abiotic factors that influence freshwater ecosystems [41]. Comparison of the latitudinal richness patterns of Mesozoic and modern turtles strongly suggests that today's pattern is one that became established early in testudinate history, potentially as early as the Jurassic, and the limited geographical shifts we observe may reflect extended niche conservatism through time [15,47–51]. Moreover, climate appears to have had a profound influence on turtle distributions and may account for the asymmetrical global distribution of the turtle LBG around the equator, in the Mesozoic at least.

## Supplementary Material

File name: Nicholson_et_al_Dataset_1_ESM.xlsx Title: Dataset 1Description: Mesozoic turtle occurrence data downloaded from http://fossilworks.org and associated data summaries used in analyses

## Supplementary Material

File name: Nicholson_et_al_Dataset_2_ESM.xlsx Title: Dataset 2Description: Calculations for palaeo-non-marine area at 15 degree palaeolatitudinal bands for Jurassic, Lower Cretaceous and Upper Cretaceous time bins used in GLS analyses

## Supplementary Material

File name: Nicholson_et_al_Mesozoic_latitude_script_ESM.R Title: Mesozoic turtle latitude analyses R scriptDescription: R script for cleaning and partitioning Mesozoic turtle occurrence data into 15 degree palaeolatitudinal bands and various temporal bins, Shareholder Quorum Subsampling and generalised least squares regression analyses.

## Supplementary Material

File name: Nicholson_et_al_manual_splits_9Myr_splits_ESM Title: Time binsDescription: File containing temporal bin information for use in Shareholder Quorum Subsampling analyses

## Data accessibility

The datasets and code supporting this article have been uploaded as part of the electronic supplementary material.
